# How to Disguise the Bitter Taste of Quinine

**Published:** 1877-10

**Authors:** Lester Curtis

**Affiliations:** 806 Wabash Avenue


### How to Disguise the Bitter Taste of Quinine.

*To the Editor of the* Journal and Examiner.

Some substance which would effectually disguise the taste of quinine and other bitter medicines, has been a great desideratum. Such a substance has been found in glycyrrhizine, which is merely the sweet principle of liquorice, rendered soluble by ammonia. An elixir containing this substance has been used, which is prepared according to the following formula:

| | | |
|---|---|---|
| ℞ | Powdered coriander, } Powdered caraway, } | aa. gr. cviij |
| | Powdered cinnamon, | gr. xciij |
| | Powdered star anise, } Powdered tonqua, } | aa. gr. lxij |
| | Powdered canilla, } Powdered nutmegs, } Powdered cloves, } | aa. gr. xxxj |
| | Ammoniacal glycyrrhizine, | ℥j gr.cxl |
| | Oil of orange, fresh, | gr. xxxi |
| | Alcohol, | f℥ xvj |
| | Water, | f℥ xvj |
| | Syrup, | f℥ xlviij |

The aromatics are placed in a percolator and exhausted with a menstruum composed of the oil of orange, alcohol and water; the percolate is mixed with the syrup, the glycyrrhizine, dissolved in a small quantity of boiling water, is mixed with the liquid, and water added to make the whole mixture five pints.

The method of using the elixir is to pour out a small quantity, say a dessert-spoonful, add the quinine in powder, mix and swallow, and then follow with a small quantity of the elixir.

This prescription is complex, cannot be prepared extemporaneously, will mask only a small quantity of the quinine, and offers no advantages over the glycyrrhizine in substance. If a small quantity of glycyrrhizine be taken in the mouth, quinine can be taken afterwards without perceiving the slightest bitter taste, the drug is masked perfectly, it is as tasteless as chalk, and not nearly so disagreeable to take; nor does any disagreeable taste return in the mouth. A very large dose of quinine may be covered in this way.

It is a very great convenience to combine the substances so that they may be given in one dose. This may be accomplished by suspending the quinine in a solution of the glycyrrhizine. A grain of glycyrrhizine will cover the taste of a grain of quinine, and two grains of glycyrrhizine will dissolve in a drachm of syrup. I usually make a mixture, consisting of fifteen grains each of glycyrrhizine and sulphate of quinine, in an ounce of simple syrup. This mixture will give nearly two grains of quinine to the drachm, and in it the taste of the quinine is almost perfectly disguised; there is a slight trace of bitter, but it is not at all disagreeable, and leaves the mouth in a short time. This mixture is a tolerably permanent one; the quinine will not settle to the bottom for two or three days, and when it does settle, it may be shaken up and used nearly as well as though freshly prepared.

Children and fussy women take this mixture not only without objecting, they even like it.

If it is desired to use a larger proportion of quinine to the ounce, the syrup must be mixed with water in order to dissolve the larger quantity of glycyrrhizine necessary to cover it. I have used as much as thirty grains of quinine to the ounce of the mixture, giving nearly four grains to the drachm. The disguise, however, is not as complete as in the other mixture; and, as a considerable quantity of water must be used, the quinine is not held in suspension as well.

LESTER CURTIS, M. D.

806 Wabash Avenue.

